# Mortality risk factors among National Football League players: An analysis using player career data

**DOI:** 10.12688/f1000research.21235.3

**Published:** 2020-09-10

**Authors:** Justin Ehrlich, Brittany Kmush, Bhavneet Walia, Shane Sanders

**Affiliations:** 1 Sport Analytics, Syracuse University, Syracuse, NY, 13244, USA; 2 Public Health, Syracuse University, Syracuse, NY, 13244, USA

**Keywords:** CTE, concussions, football, gridiron football, NFL, chronic traumatic encephalopathy, sports

## Abstract

In general, National Football League (NFL) players tend to live longer than the general population. However, little information exists about the long-term mortality risk in this population. Frequent, yet mild, head trauma may be associated with early mortality in this group of elite athletes. Therefore, career playing statistics can be used as a proxy for frequent head trauma. Using data from Pro Football Reference, we analyzed the association between age-at-death, position, and NFL seasons-played among 6,408 NFL players that were deceased as of July 1, 2018. The linear regression model allowing for a healthy worker effect demonstrated the best fit statistics (F-statistic = 9.95, p-value = 0.0016). The overall association of age-at-death and seasons-played is positive beginning at the 10.75 and 10.64 seasons-played point in our two models that feature seasons-played and seasons-played squared as explanatory variables. Previous research that does not account for this survivorship bias/healthy worker effect may not adequately describe mortality risk among NFL players.

## Introduction

Very little information exists about mortality and long-term health outcomes among National Football League (NFL) players. Elite football players tend to have a lower overall mortality rate than the general population, often attributed to routine physical activity ^1, 2^. However, this occupational group cannot be directly compared to the general population ^3^. Several studies in small numbers of NFL players have found an association between traumatic brain injuries with depression, suicide, dementia, and chronic traumatic encephalopathy ^4– 6^. There is mounting evidence that even sub-clinical head impacts, especially when they occur frequently, can also lead to these adverse health outcomes ^7, 8^. However, these relationships are difficult to study systematically due to few cases, challenges with diagnostics, and long lag time from the injury to symptom onset. Yet, there exists a rich repository of data surrounding NFL career playing statistics ^9^. We hypothesize that certain player career attributes, including position-of-play and seasons-played, are likely to be strong predictors for mortality from repeated, yet mild, head trauma. Here, we study the association between mortality and NFL seasons-played, while controlling for playing position. 

## Methods

Data was collected from Pro Football Reference, a free online database maintained by Sports Reference LLC that includes playing statistics from every player in NFL history, over 25,000 in total, with meticulously recorded data beginning in 1922 ^9^. Variables of interest include birthdate, death date, position, height, weight, and seasons-played. This data is freely and publicly available from Pro Football Reference ^9^. Individuals with any missing data were eliminated, leaving 24,740 players. Of those, 6,408 (25.9%) had died according to Pro Football Reference, as of July 1, 2018. Height and weight were used to calculate the players’ Body Mass Index (BMI) by dividing weight (kg) by height (m) squared ^10^. Playing position was divided into three standard categories according to previous literature ^11^. As this is a complete census of the deceased players, we retained outliers as to not introduce selection bias. To address outliers, we specified robust standard errors to measure risk factors for mortality in a manner consistent with valid derivation of t-statistics.

Category 1: defensive back, quarterback, wide receiver, and kicker: 1,600 dead/8,415 players (19%).

Category 2: running back, linebacker, tight end: 1,690 dead/7,228 players (23%).

Category 3: offensive and defensive linemen: 3,118 dead/9,097 players (34%).

### Statistical analysis

Expected age-at-death was calculated from the 2017 National Vital Statistics Report ^12^ using average years of life remaining at 20 years of age for the decade of the 20th year plus 20. Age-at-death residuals were calculated as observed age-at-death minus expected age-at-death. This analysis was completed in Stata Version 14 ^13^, and data was visualized using R 3.6.1 ^14^. Associations were assessed using linear regression models with a quadratic term for seasons-played. Specifically, we use (position) fixed-effect ordinary least squares modeling to determine whether associations exist between age-at-death residual, number of NFL seasons-played (squared), and position category fixed effects. In these models, we seek to assess whether career duration exposure relates significantly to age-at-death residual conditional on position-of-play. The survivorship bias turning point was calculated using standard differential calculus techniques (i.e., calculating the minimum point of a best fit surface). 

**Base Model:**

*Age at Death Residual*_*i,t*_ = *β*_0_ + *β*_1_ *Number of Seasons Played*_*i,t*_ + *ε*_*i,t*_

**Seasons-played Squared Model:**

*Age at Death Residual*_*i,t*_ = *β*_0_ + *β*_1_ *Number of Seasons Played*_*i,t*_ + *ε*_*i,t*_ + *β*_2_ *Number of Seasons Played* ^2^_*i,t*_ + *ε*_*i,t*_

**Position Category Fixed Effects Model**

*Age at Death Residual*_*i,t*_ = *β*_0_ + *β*_1_ *Number of Seasons Played*_*i,t*_ + *ε*_*i,t*_ + *β*_2_ *Number of Seasons Played* ^2^_*i,t*_ + *ε*_*i,t*_ + *β*_3_ *Position Category*_*i*_ + *ε*_*i,t*_

## Results and discussion

Table 1 indicates substantial demographic sample variation between players of different position categories in height, weight, BMI, and age-at-death. Figure 1a– Figure 1b indicate a possible survivorship bias among players of Category I and II. Certain healthy or durable players can play an increased number of seasons without a corresponding reduction in expected age-at-death as compared to players of shorter career duration ^3^.

**Table 1.** Demographics of deceased National Football League (NFL) players (1922–2018).

Characteristic	Total	Category 1 Players	Category 2 Players	Category 3 Players
N	6408	1600	1690	3118
Median Year of Birth (Range)	1919 (1876–1992)	1919 (1883–1992)	1922 (1880–1992)	1917 (1876–1986)
Average Age-at-death (sd) (years)	69.1 (15.8)	69.5 (15.8)	68.0 (16.4)	69.6 (15.3)
Median Year of Death (Range)	1992 (1923–2018)	1993 (1925–2018)	1996 (1924–2018)	1990 (1923–2018)
Median Seasons Played (IQR)	2 (3)	3 (4)	2 (4)	2 (3)
BMI (sd) (kg/m ^2^)	27.6 (2.73)	25.8 (1.55)	27.4 (2.19)	28.6 (2.97)
Height (sd) (cm)	184 (6.04)	181 (5.40)	183 (5.68)	186 (5.94)

BMI – body mass index

**Figure 1a.**
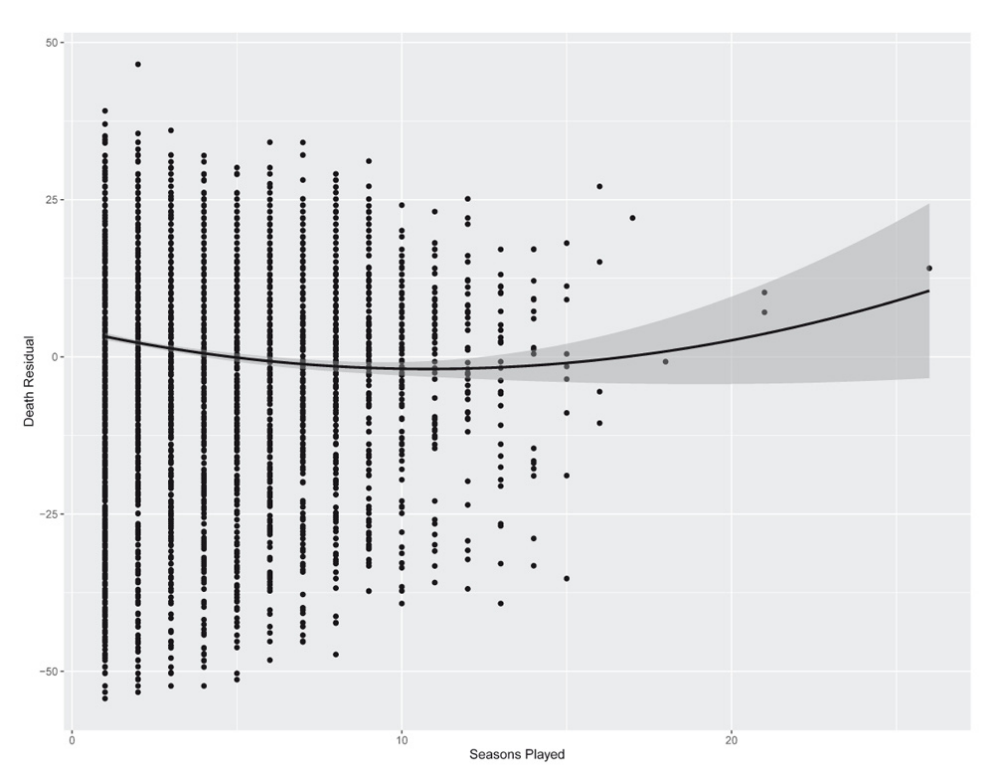
Age-at-death residual versus seasons-played of deceased National Football League (NFL) players (1922–2018) N=6408. Dots represent individual players; Solid line represents a quadratic trend.

**Figure 1b.**
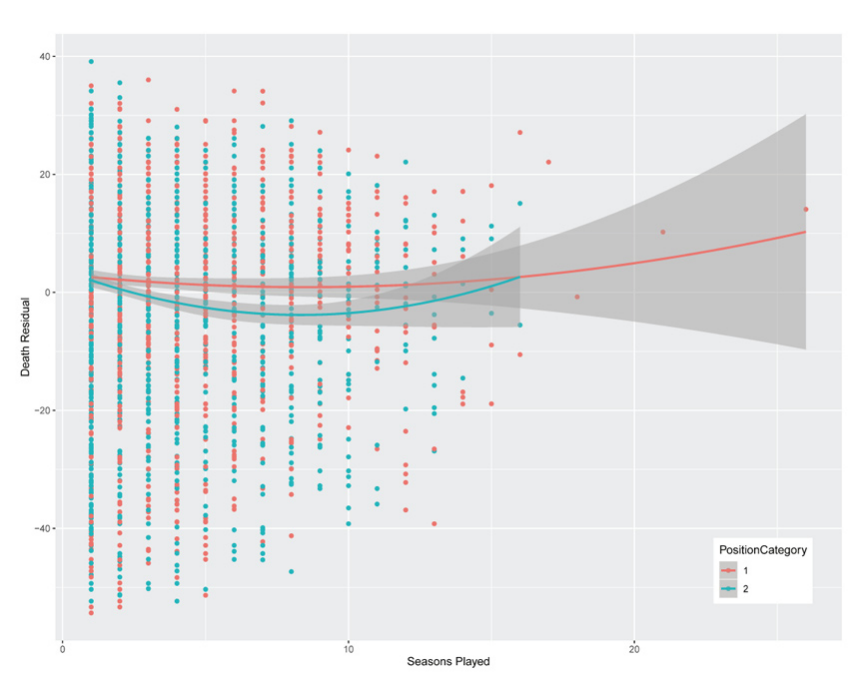
Age-at-death residual versus seasons-played for category 1 and 2 deceased National Football League (NFL) players (1922–2018) N=3,290. Dots represent individual players; Solid line represents a quadratic trend.

The *Seasons-played Squared* and *Position Category Fixed Effects* models specify a quadratic term for number of NFL seasons-played. For both models, the coefficient for this variable is significant and improves the model’s explanatory power according to an Anova F-test for difference in overall model significance (F-statistic = 9.95, p-value = 0.0016; F-statistic=10.98, p-value<0.001) ( Table 2). We calculate that overall association of age-at-death residual and seasons-played is positive beginning at 10.75 and 10.63 seasons-played for the *Seasons-played Squared* and *Position Category Fixed Effects* model, respectively. This demonstrates a survivorship effect within the NFL population, where certain players are not as prone to play-related mortality risk. We define this effect within the NFL population as a longitudinal survivorship bias where certain players’ ability to play diminishes over time such that the players are removed from the cohort. For these deceased players, the survivorship bias is sufficiently strong to dominate an observed mortality risk, where the survivorship effect drives the negative relationship between seasons-played and age-at-death residual for those playing fewer than 10.75 (10.63) seasons. The survivorship bias and the mortality risk hold conditional upon position category control variables, as found in previous literature ^11^. However, dividing players into three position categories may not sufficiently capture the differing on-field exposures that may contribute to mortality.

**Table 2.** Linear regression models predicting age-at-death Residuals among National Football League (NFL) players (1922–2018) N=6408.

	Base			Seasons-played Squared			Position Category Fixed Effects		
*Predictors*	*Estimates*	*Standard* *Error*	*p*	*Estimates*	*Standard* *Error*	*p*	*Estimates*	*Standard* *Error*	*p*
(Intercept)	3.402	0.315	**<0.001**	4.337	0.433	**<0.001**	4.957	0.473	**<0.001**
Seasons-played	-0.562	0.073	**<0.001**	-1.161	0.203	**<0.001**	-1.169	0.203	**<0.001**
Seasons-played Squared				0.054	0.017	**0.002**	0.055	0.017	**0.001**
Position Category 1							-0.042	0.515	0.934
Position Category 2							-2.277	0.504	**<0.001**
Position Category 3							Reference	--	**--**
Observations	6408			6408			6408		

## Policy implications

This study suggests that NFL career duration is typically a risk factor for early mortality. However, player characteristics leading to extreme career survivorship are also important and can act to countervail the risk exposures from NFL seasons played. Injury histories of players with a relatively short NFL career may be particularly important toward recommending modifications to game play that are conducive to mitigating these early mortality risk factors. We also find variation in early mortality risk by position category. Again, rule changes that serve to mitigate risks (e.g., head impact) at particularly vulnerable positions may lead to marked long term improvements in player health. 

## Conclusion

This paper finds evidence of both player health risk (in terms of age-at-death residual) for increasing NFL seasons played and a survivorship bias among NFL players. For Category I and II players, the latter risk dominates the former for NFL players with sufficient career survivorship. This effect holds conditional upon position-of-play control variables. Previous research not accounting for this survivorship bias/healthy worker effect may not adequately describe mortality risk among NFL players.

## Future work

As this study only used publicly available data, we only analyzed all-cause mortality as cause of death is not included in the database. Both cause of death and quality of life throughout life are very important to the study of the hazards associated with football. We are pursuing additional research to examine the association of on-field playing characteristics with mortality and cause of death among NFL players.

## Ethics

This study was determined by the Syracuse University Institutional Review Board to not be human subjects research and therefore, not to require review and oversight.

## Data availability

### Underlying data

Variables of interest: birthdate, death date, position, height, weight, and seasons-played were freely and publicly available from Pro Football Reference ^9^, and was collected on July 1st, 2018. Height and weight were then used to calculate the players’ Body Mass Index (BMI) by dividing weight (kg) by height (m) squared ^10^.

Data are available under the terms of the Creative Commons Zero "No rights reserved" data waiver (CC0 1.0 Public domain dedication).
