# Supplementary material for: Mortality risk factors among National Football League players: An analysis using player career data
Source: F1000Res. 2020 Sep 10;8:2022. Originally published 2019 Nov 28. [Version 3] doi: 10.12688/f1000research.21235.3 (PMC7406953; doi:10.12688/f1000research.21235.3)
Supplement: Peer review history

# Peer review: Reviewer response for version 3

**DOI:** 10.5256/f1000research.29355.r71171

**Published:** 2020-09-10

**Authors:** Adam M. Finkel

**Affiliations:** 1 Department of Environmental Health Sciences, University of Michigan School of Public Health, Ann Arbor, MI, USA

Not sure if this comment is necessary, but the authors have made a technical amendment to the final version-- I certainly "approve" the amendment, as I approved the revised ms. some weeks ago.

 [I would still welcome comment on the points I raised in my re-review, but not expecting this, as the points are relatively minor].

Is the work clearly and accurately presented and does it cite the current literature?

Partly

If applicable, is the statistical analysis and its interpretation appropriate?

Partly

Are all the source data underlying the results available to ensure full reproducibility?

Yes

Is the study design appropriate and is the work technically sound?

Partly

Are the conclusions drawn adequately supported by the results?

Partly

Are sufficient details of methods and analysis provided to allow replication by others?

Partly

Reviewer Expertise:

quantitative risk assessment; epidemiology; regulatory policy

I confirm that I have read this submission and believe that I have an appropriate level of expertise to confirm that it is of an acceptable scientific standard.

# Peer review: Reviewer response for version 2

**DOI:** 10.5256/f1000research.28090.r68307

**Published:** 2020-08-05

**Authors:** Adam M. Finkel

**Affiliations:** 1 Department of Environmental Health Sciences, University of Michigan School of Public Health, Ann Arbor, MI, USA

This version is improved from the original, and I have no remaining “reservations.”  However, I do have a very few comments:

  

1. The authors recognize that what they may have found is a “longitudinal survivorship effect” within a worker cohort, not a “healthy worker effect,” and they now use the former term more often than the latter. However, the first time either term appears is in the Abstract, where “healthy worker effect” alone is used. Because so many articles in the football-risk literature fail to appreciate the HWE, it’s important that this one—which does “get it”—uses the term correctly.
2. I may not have been completely clear in my prior review about my concerns with respect to outliers. The authors have justified their use of players with an extremely long number of seasons played as a *statistical* matter (although their response to reviews claims they have added a statement about the limitations of using all data, which they haven’t really done…). But my concern was/is about how the outliers with respect to the number of *seasons* may not in fact be outliers with respect to the number of *games played, *and thus that the entire analysis using seasons may be sub-optimal. I merely note that the five players with the greatest number of seasons (Tittle, Baugh, Blanda, Unitas, and Morrall) may well not have played any more *games* than players in the middle of the distribution, because they were backup QBs for part of their careers. So a second analysis using games rather than seasons as the main independent variable could be very illuminating.
3. I agree that the authors have examined all-cause mortality, but that’s why it’s slightly confusing that they invoke “frequent head trauma” in the abstract and use three keywords relating to concussion and CTE. I look forward to their future research where cause-of-death and quality-of-life data may be marshaled to firm up the connection between seasons (games) played, head trauma, and *those* causes of death and morbidity that are plausibly related to head trauma.
4. The new “policy implications” paragraph is welcome, but it does not mention the single most important point, one that the authors put in their response to reviews: “To make the game safer, we must identify which players are at an elevated risk.” *This *is the policy implication of a survivorship effect. As a policy analyst, I would personally go on to say that we need not only to identify those players but craft policies (rule changes, medical monitoring, protective equipment, etc.) that can reduce these players’ risks without discriminating against them needlessly because of their “susceptibility.” The football literature is already replete with confused and tautologous statements about how CTE only occurs “among those who are sensitive.”

Is the work clearly and accurately presented and does it cite the current literature?

Partly

If applicable, is the statistical analysis and its interpretation appropriate?

Partly

Are all the source data underlying the results available to ensure full reproducibility?

Yes

Is the study design appropriate and is the work technically sound?

Partly

Are the conclusions drawn adequately supported by the results?

Partly

Are sufficient details of methods and analysis provided to allow replication by others?

Partly

Reviewer Expertise:

quantitative risk assessment; epidemiology; regulatory policy

I confirm that I have read this submission and believe that I have an appropriate level of expertise to confirm that it is of an acceptable scientific standard.

# Peer review: Reviewer response for version 2

**DOI:** 10.5256/f1000research.28090.r68308

**Published:** 2020-07-30

**Authors:** Thomas Sadler

**Affiliations:** 1 Department of Economics and Decision Sciences, Western Illinois University, Macomb, IL, USA

Very good updates.

Is the work clearly and accurately presented and does it cite the current literature?

Yes

If applicable, is the statistical analysis and its interpretation appropriate?

Yes

Are all the source data underlying the results available to ensure full reproducibility?

Yes

Is the study design appropriate and is the work technically sound?

Yes

Are the conclusions drawn adequately supported by the results?

Yes

Are sufficient details of methods and analysis provided to allow replication by others?

Yes

Reviewer Expertise:

Economics, Environment,  Professional Sports, Pandemics

I confirm that I have read this submission and believe that I have an appropriate level of expertise to confirm that it is of an acceptable scientific standard.

# Peer review: Reviewer response for version 1

**DOI:** 10.5256/f1000research.23382.r63530

**Published:** 2020-06-03

**Authors:** Thomas Sadler

**Affiliations:** 1 Department of Economics and Decision Sciences, Western Illinois University, Macomb, IL, USA

This research analyzes the impact of the number of years played in the NFL and position on life expectancy. The question is excellent.  The model concisely addresses the question, and the data are comprehensive. This is an important research study that could be replicated for other professional sports. An additional study could break the regressions down by individual position, instead of three categories, to see if there is a further distinction. But that is not necessary for this paper.

Is the work clearly and accurately presented and does it cite the current literature?

Yes

If applicable, is the statistical analysis and its interpretation appropriate?

Yes

Are all the source data underlying the results available to ensure full reproducibility?

Yes

Is the study design appropriate and is the work technically sound?

Yes

Are the conclusions drawn adequately supported by the results?

Yes

Are sufficient details of methods and analysis provided to allow replication by others?

Yes

Reviewer Expertise:

Economics, Environment,  Professional Sports, Pandemics

I confirm that I have read this submission and believe that I have an appropriate level of expertise to confirm that it is of an acceptable scientific standard.

# Peer review: Reviewer response for version 1

**DOI:** 10.5256/f1000research.23382.r57258

**Published:** 2019-12-16

**Authors:** Adam M. Finkel

**Affiliations:** 1 Department of Environmental Health Sciences, University of Michigan School of Public Health, Ann Arbor, MI, USA

This was a frustrating paper to review: on the one hand, I definitely applaud any analysis of athletes that compares athletes TO athletes and thus avoids the healthy worker effect HWE). (See Nguyen *et al. *2019 ^1^ for a good recent example of avoiding this pitfall; see footnote 2 in this article for a good put-down of the HWE fallacy ^2^). On the other hand, I don’t think this analysis actually involves the HWE *at all*; as I will discuss, it actually purports to find a “frailty” issue *within* this worker population, which is a very different phenomenon and one with rather different research and policy implications than a true HWE finding.

 In this context, a true HWE occurs when reach the mistaken conclusion that X is not riskier than not-X (as in “football players don’t die more often of cancer than general population”), or even that X is safer than not-X, but what’s really happening is that *being in class X* shows you were less likely to have the risk than general to begin with. This is important to find and point out, because then you either have to adjust for it (see Choi *et al.* ^3^) or do a different study that compares apples to apples. But what these researchers may have found (see below) is that *within* subpopulation of NFL players, some are less frail than others. This is heterogeneity-dynamics at work (see Manton, K.G., E. Stallard, and J.W. Vaupel. 1986. Alternative models for the heterogeneity of mortality risks among the aged. J. Am. Stat. Assoc. 81:635-644 ^4^). Heterogeneity-dynamics means that you may need to adjust the slope of an observed dose-response function to account for the fact that there are 2 or more subpopulations *within* the subpopulation—everyone can have an individual positive association between seasons and risk, but the *group* may have a flat (null) slope simply because those who played the most seasons were not “lucky” but more *immune.*

 The difference between a finding of an HWE between football and the general public, versus the finding of two of more differentially-susceptible subgroups *within* the NFL population, is far from merely semantic, because the practical implications of the two situations are so different. Finding an HWE allows the researcher to *correct *for it, either by discarding a flawed analysis in favor of an apples-to-apples comparison, or by taking the existing analysis and trying to re-estimate the odds ratio (see, for example, Joffe, 2012 ^5^). Even without correction, researchers who appreciate the pitfalls of the HWE can simply say that it leads to *false negatives* preferentially: comparing, for example, the cognitive performance of 60-year-old retired NFL players against the general population of that age would tend to divert attention away from the consequences of repeated head trauma (RHT) simply because the general population includes many persons who were never fit enough to work, let alone work in this taxing occupation.

 But finding a negative-then-positive relationship between length of (NFL) career and age at death, *because* the cohort under study has, *by definition, *more fit persons remaining as more and more of the cohort dies early, may have no empirical or policy implications whatsoever. The authors say nothing about how we might even identify who among incoming NFL players might “benefit” from longer careers and whose dose-response is the most steeply negative—if we could, and IF we had the means and the will to discourage the latter group from choosing this occupation, THEN perhaps we could make use of the “finding” that some players are not at risk to the extent that others are (there is an extensive literature about the gap between identifying a powerful interindividual risk factor in a working population—these are usually genetic factors, such as “slow acetylators” who are more at risk from certain occupational chemicals—and the wisdom of trying to exclude these people from the workplace. Generally, policy analysts prefer interventions that can make the workplace safe for *everyone* who participates—imagine a policy of not allowing people with hemophilia to become carpenters, as opposed to OSHA regulations that mandate guards on saws so that no one will be cut by them). 

 So this article, at most, finds an “expected curiosity”—that once you have had a long career, you probably are revealing to an epidemiologist that you have been more “immune” to the harmful effects of that career than the average person—and then says nothing about how we could use the finding to adjust scientific conclusions or policy responses. By invoking the HWE throughout the paper, the authors are not only using the wrong term, but inviting statistical corrections or policy responses that have already been made or that would not respond to what they may have actually found.

 But all the foregoing assumes that the authors have actually found evidence of a “resistant subpopulation” within the NFL cohort, and I’m not sure that’s the case. The authors don’t mention alternative explanations for the slight upslope in Figures 1a and 1b, including: (1) reverse causation—if a significant number of players died on the field or soon after sustaining football-related injuries early in their careers, then of course the remaining population would not have “negative death residuals” that large; and (2) effect modification—similarly, if physical inactivity leads to earlier death, then players who sustained career-ending injuries early on would die earlier than others.

 More problematic is the use of rote statistics without also applying “common sense” to the finding. How robust, in particular, is the slight upslope obtained by regression to the presence of *outliers*? The interactive Figures show that the five players with the longest careers were Sammy Baugh, Johnny Unitas, George Blanda, Earl Morrall, and YA Tittle. Some of these (Morrall) I believe played long careers but were backups much of the time, so an index based on number of *games* rather than seasons might have shown something different. It would be important to explore what happens to the upslope if outliers were trimmed—I say this in part because visual exploration of the Figures does not present a compelling “common sense” picture of a positive slope among the longer careers; I believe the numbers, but the visual impression, especially excluding outliers, is one of a rather FLAT dose-response that one might be convinced is slightly negative and then very slightly positive. Similarly, I accept (p. 3) that the quadratic model fits slightly better than the linear one, but the authors don’t let on how well the linear model fit in the first place. Could they be “finding” something more sophisticated simply by over-fitting? Also, I dispute that the fixed-effects model is correct, with respect to the kinds of effects (CTE, dementia) the authors clearly are trying to shed light on. The three categories they use seem out-of-place here: other studies have shown that the three positions with the greatest cumulative amount of RHT (instances times g-forces) are tight ends, quarterbacks, and defensive linemen, and yet these are in three *different* categories in this model!

 Finally, the authors focus on mortality, which is fine, but completely ignore quality of life. Just consider the case of Earl Morrall, who lived to age 79 (death residual of > 10) but who reportedly had Stage 4 CTE and a reduced QOL. Someone who comes away from the paper concluding that “once you play 11 seasons, you may live longer than your cohort” may not realize that age at death is not the most relevant outcome…

 In summary, the authors should replace “HWE” with “interindividual variability in susceptibility,” explore the implications of THAT analysis, consider how robust their analyses of statistical significance actually are, and ground this paper in terms of the other more ambitious studies that have already explored the relationship between *better* indices of lifetime intensity of play and mortality/morbidity (especially Montenigro *et al.*, 2017 ^6^). They might also consider this recent paper by Mez *et al.* ^7^ and explore if and how their quadratic model might better explain (or be contradicted by) these prior findings.

Is the work clearly and accurately presented and does it cite the current literature?

Partly

If applicable, is the statistical analysis and its interpretation appropriate?

Partly

Are all the source data underlying the results available to ensure full reproducibility?

Yes

Is the study design appropriate and is the work technically sound?

Partly

Are the conclusions drawn adequately supported by the results?

Partly

Are sufficient details of methods and analysis provided to allow replication by others?

Partly

Reviewer Expertise:

quantitative risk assessment; epidemiology; regulatory policy

I confirm that I have read this submission and believe that I have an appropriate level of expertise to confirm that it is of an acceptable scientific standard, however I have significant reservations, as outlined above.

## References

[1] : Mortality Among Professional American-Style Football Players and Professional American Baseball Players. *JAMA Netw Open*.2019;2(5) : 10.1001/jamanetworkopen.2019.4223 e194223 10.1001/jamanetworkopen.2019.4223 31125098PMC6632140

[2] : A quantitative risk assessment for chronic traumatic encephalopathy (CTE) in football: How public health science evaluates evidence. *Human and Ecological Risk Assessment: An International Journal*.2019;25(3) : 10.1080/10807039.2018.1456899 564-589 10.1080/10807039.2018.1456899

[3] : Mathematical procedure to adjust for the healthy worker effect: the case of firefighting, diabetes, and heart disease. *J Occup Environ Med*.2001;43(12) : 10.1097/00043764-200112000-00007 1057-63 10.1097/00043764-200112000-00007 11765676

[4] : Alternative models for the heterogeneity of mortality risks among the aged. *J Am Stat Assoc*.1986;81(395) : 10.1080/01621459.1986.10478316 635-44 10.1080/01621459.1986.10478316 12155405

[5] : Structural Nested Models, G-Estimation, and the Healthy Worker Effect. *Epidemiology*.2012;23(2) : 10.1097/EDE.0b013e318245f798 220-222 10.1097/EDE.0b013e318245f798 22317805

[6] : Cumulative Head Impact Exposure Predicts Later-Life Depression, Apathy, Executive Dysfunction, and Cognitive Impairment in Former High School and College Football Players. *Journal of Neurotrauma*.2017;34(2) : 10.1089/neu.2016.4413 328-340 10.1089/neu.2016.4413 PMC522053027029716

[7] : Duration of American Football Play and Chronic Traumatic Encephalopathy. *Ann Neurol*.2020;87(1) : 10.1002/ana.25611 116-131 10.1002/ana.25611 31589352PMC6973077

# Response

**Published:** 2020-01-29

**Authors:** Justin Ehrlich

**Affiliations:** Syracuse University, USA

Thank you for these important points; they will lead to improvements in our paper. This was a very helpful review even though it started off in a negative sense.

We will change the way the HWE is described with respect to the data/results.  Specifically, we will frame the HWE as a longitudinal survivorship effect where people are removed from the cohort.  We agree there are heterogeneity dynamics at work and will term this as a “survivorship effect” throughout.   

We thank the reviewer for suggestions regarding the policy implications and will clarify our policy section accordingly.  To make the game safer, we must identify which players are at an elevated risk. 

While the reviewer has valid points about outliers, we would prefer to keep them in our analysis. To address outliers, we specified robust standard errors to measure risk factors for mortality in a manner consistent with valid derivation of t-statistics. We disagree with the reviewer and prefer to keep outliers in the dataset. We did not eliminate the outliers so as not to introduce selection bias.  Furthermore, this a complete census of the players and we calculated population parameters, not sample statistics; therefore, we prefer to keep all players in the analysis. We will be more clear in the revised draft that we are analyzing the population of NFL players. As such, there is no need to worry about outliers that have an unrepresentative influence vis-à-vis the underlying population. However, we will add in a statement in the limitations about our choice to keep the outliers.

We agree that quality of life and cause of death are important considerations. Here, we analyze all-cause mortality, not CTE specific mortality. Therefore, the papers the reviewer suggested may not be appropriate because we have all deaths in this population, not a selected sub-sample. While in our future research, we hope to focus on quality of life and cause-specific mortality, that is not possible with this data. We will add in statements about these limitations and future directions.
